# The Specific Agent of Typhoid Fever

**Published:** 1881-02

**Authors:** 


### THE SPECIFIC AGENT OF TYPHOID FEVER.

PROF. KLEBS, of Prague, believes that he has discovered the micro-organism which constitutes the specific agent of typhoid fever, and develops his views in a paper, published in the *Archiv fuer Experimentale Pathologie*, t. xii, p. 231, 1880. Professor Klebs has for a long time, assisted by his pupils, been making researches in this direction. He writes that he has been able to find, at the necropsy of twenty-four persons carried off by dothinenteritis microbes in various organs; in the intestinal mucous membrane, in the thickness of the cartilages of the larynx, in the pia mater, in foci of lobular pneumonia, in the mesenteric ganglia, in the parenchymata of the liver, and generally diffused in the organs which showed the most decided lesions. These micro-organisms showed themselves in the form of rods, about eighty micrometers in length and 0.5 to 0.6 micrometers in thickness. They have been constantly observed in the bodies of dothinenteric patients since the attention of Professor Klebs was drawn to the subject, and they are always absent from the organs, and specially the intestines, of subjects who have died from any other disease than typhoid.

